# iTRAQ-Based Quantitative Analysis of Responsive Proteins Under PEG-Induced Drought Stress in Wheat Leaves

**DOI:** 10.3390/ijms20112621

**Published:** 2019-05-28

**Authors:** Yajing Wang, Xinying Zhang, Guirong Huang, Fu Feng, Xiaoying Liu, Rui Guo, Fengxue Gu, Xiuli Zhong, Xurong Mei

**Affiliations:** 1 Institute of Environment and Sustainable Development in Agriculture, Chinese Academy of Agricultural Sciences, 100081 Beijing, China; wyj8664@126.com (Y.W.); zhxyhb516@126.com (X.Z.); hguirong0920@126.com (G.H.); 82101176076@caas.cn (F.F.); liuxiaoying@caas.cn (X.L.); guorui01@caas.cn (R.G.); gufengxue@caas.cn (F.G.); 2 State Engineering Laboratory of Efficient Water Use and Disaster Mitigation for Crops/Key Laboratory for Dryland Agriculture of Ministry of Agriculture, 100081 Beijing, China

**Keywords:** proteomics, wheat, drought, leaf, iTRAQ

## Abstract

Drought is an important abiotic stress that seriously restricts crop productivity. An understanding of drought tolerance mechanisms offers guidance for cultivar improvement. In order to understand how a well-known wheat genotype Jinmai 47 responds to drought, we adopted the iTRAQ and LC/MS approaches and conducted proteomics analysis of leaves after exposure to 20% of polyethylene glycol-6000 (PEG)-induced stress for 4 days. The study identified 176 differentially expressed proteins (DEPs), with 65 (36.5%) of them being up-regulated, and 111 (63.5%) down-regulated. DEPs, located in cellular membranes and cytosol mainly, were involved in stress and redox regulation (51), carbohydrate and energy metabolism (36), amino acid metabolism (24), and biosynthesis of other secondary metabolites (20) primarily. Under drought stress, TCA cycle related proteins were up-regulated. Antioxidant system, signaling system, and nucleic acid metabolism etc. were relatively weakened. In comparison, the metabolism pathways that function in plasma dehydration protection and protein structure protection were strongly enhanced, as indicated by the improved biosynthesis of 2 osmolytes, sucrose and Proline, and strongly up-regulated protective proteins, LEA proteins and chaperones. SUS4, P5CSs, OAT, Rab protein, and Lea14-A were considered to be important candidate proteins, which deserve to be further investigated.

## 1. Introduction

Wheat (*Triticum aestivum* L.), the second staple human food crop, is regarded as the leading source of vegetable protein in human nutrition [1] and has been subjected to intensive breeding and selection for almost a century [2]. Drought stress is one of the main factors restricting crop productivity and limiting the distribution of species worldwide. Thus, selection efforts have been made to improve drought tolerance to ensure good yield in drought-prone areas.

Studies on molecular and physiological mechanisms of plants in response to drought stress have been extensively conducted to guide cultivar improvement. Once subjected to drought stress, the response processes begin with perception and transduction of drought signal, which usually evokes other subsequent processes. Stomatal adjustment, namely rapid stomatal closure, is triggered by an ABA increase to decrease water loss from leaves [3]. Osmolyte, such as proline, glutamate, glycine betaine and sugars (mannitol, sorbitol and trehalose), accumulate to protect protoplasm from dehydration and enzyme inactivation [4]. Antioxidant systems enhance scavenging reactive oxygen species (ROS) strongly, which attack cellular membrane and organelle through peroxidation damage. These responses involve multiple biochemical pathways and significant changes in gene expression. A large number of studies on wheat plants have identified, cloned, and characterized new genes involved in drought response [5,6,7,8].

Accelerated by high throughput technology, genomics, transcriptomics, and proteomics have been rapidly advancing, which facilitate both the elucidation of underlying mechanisms of stress tolerance as well as advancement in breeding technology. A chromosome-based draft sequence of the bread wheat genome was published by the International Wheat Genome Sequencing Consortium in 2012 [9]. It is expected to enable a more effective and focused approach to the breeding of high-yield varieties with increased stress tolerance. Recently, with improvements in sequencing, an annotated reference genome with a detailed analysis of gene content among the structural organization for all the chromosomes and subgenomes was presented by the International Wheat Genome Sequencing Consortium. Quantitative trait mapping and CRISPR-based genome modification show crucial roles in applying this genome in agricultural research and breeding [10]. Moreover, high-throughput transcriptomic studies have provided substantial quantities of data to explore mRNA levels under stresses. However, since protein functions determine the final biological processes that are involved in adaption to drought stress, the changes in gene expression levels do not correspond directly to protein expression levels, let alone the growth phenotypes in wheat, due to the post-translation modification of the protein, which cannot be detected by transcriptomics analyze. Proteomics, as a study on gene products, namely protein, enables the observation of the products of gene expression that have a physiological effect on the plant. Thus, large-scale screening of drought-responsive proteins using comparative proteomic analysis is becoming one of the best strategies to investigate the stress responses of plants. Several recent studies have attempted to describe changes in proteome in response to drought stress [4,11,12,13]. The known drought-responsive proteins are mainly involved in various metabolic pathways, ranging from regulation of carbohydrate, nitrogen, energy and redox and amino acid metabolism to antioxidant capacity, cytoskeleton stability, signal transduction, as well as mRNA, and protein processing [4,11]. By linking the differentially expressed proteins (DEPs) back to the genes, candidate genes for agronomic traits can be selected, leading to the advances of functional molecular markers for expediting and assisting crop breeding practices [14].

In the North China Plain (NCP), the main wheat production region of China, only less than 30% of the rainfall occurs in the wheat-growing season, which meets only about 25–40% of the water requirements of wheat. As a result, more than 70% of the irrigation water used is for winter wheat [15]. Irrigation usage for wheat threatens the sustainability of the groundwater resource [16]. Breeding prominent rain-fed cultivars, which are capable of actively resisting drought stress, also fully utilizing rainfall, is an important pathway to ensure good harvest stably. Jinmai 47, grown in a large area in the NCP, where proper irrigation is unavailable, is a well-known rain-fed cultivar and is also employed as a parent material in cultivar breeding. Proteomics analysis will hopefully shed light on the key response mechanism to drought stress, and offer useful information for the breeding of new rain-fed cultivars.

Two-dimensional gel electrophoresis (2-DE) and Mass Spectrometry (MS) have been adopted for many years to identify proteins. Gel-based approaches were widely used because of their simplicity and reproducibility. However, the identification of 2-DE is limited by protein abundance (proteins with low abundance are unable to be detected), narrow pI range coverage [17]. On the other hand, isobaric tags for the relative and absolute quantitation (iTRAQ) technique is a high-throughput proteomic technique with higher sensitivity that allows simultaneous identification and quantification of proteins, including low-abundant proteins, in no more than eight samples with high coverage [18,19]. The technique has been applied to *Arabidopsis thaliana* [20], *Zea mays* L. [21], *Brassica napus* [22,23], *Triticum aestivum* L. [24] and *Nicotiana tabacum* [25] in recent years. In this study, iTRAQ-based proteomics analysis, a complement to transcript analysis, was implemented to elucidate the responses of wheat cultivar Jinmai 47 to PEG-induced drought stress.

## 2. Results

### 2.1. Physiological Changes in Wheat Seedlings under Drought Stress

**Figure 1:**
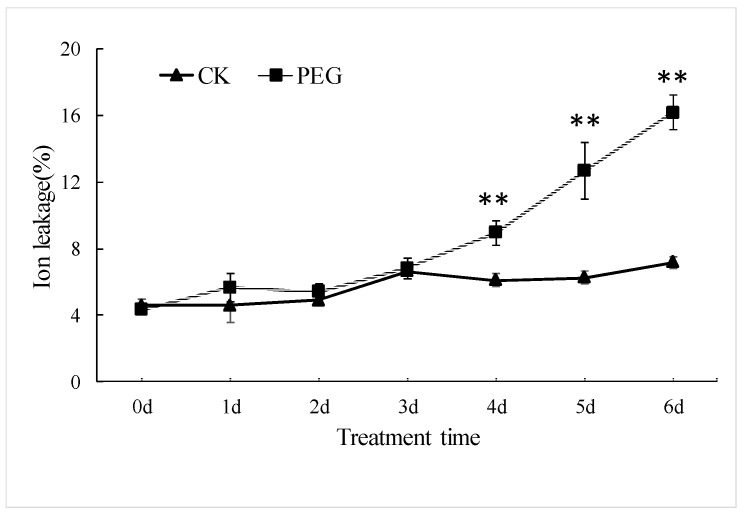
Ion leakage of wheat leaves under drought stress and well-watered condition. ** represents that there is significant differences in wheat in two treatments at *p* < 0.01. “d” in treatment time represents day here.

Stress severity depends on both stress intensity and stress time. Seedlings of Jinmai 47 were constantly treated with a high concentration of 20% of PEG solution, which means that due to drought severity the seedlings’ suffering increased over time. Ion leakage of cellular membrane closely relates to its integrity and stability, and is thus frequently used as a stress indicator. The membrane ion leakage demonstrated an increasing trend with stress time prolonged. It became significantly higher than controls (CK) after 4 days of stress and drastically increased after 5–6 days, reaching 125.6% higher than that of CK (*p* < 0.01) after 6 days of stress (Figure 1). The drastic permeability increase predicted severe irreversible damage to the cellular membrane, as well as plant tissues when stress continued for 5–6 days. However, 4 days of stress is considered to evoke a strong resistant response.

### 2.2. Identification of Proteins in Response to Drought Stress

**Figure 2:**
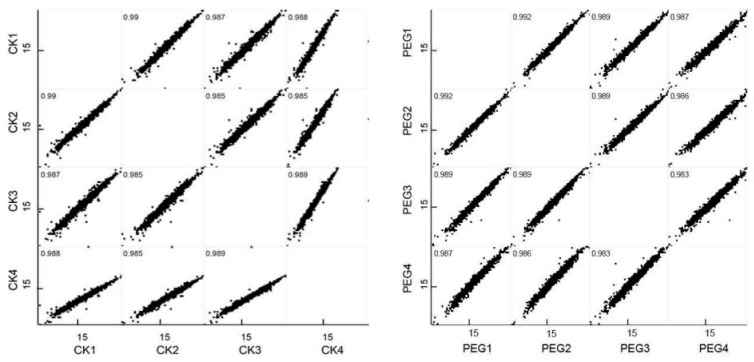
Correlations of replicates of wheat leaves under drought stress and well-watered condition.

**Figure 3:**
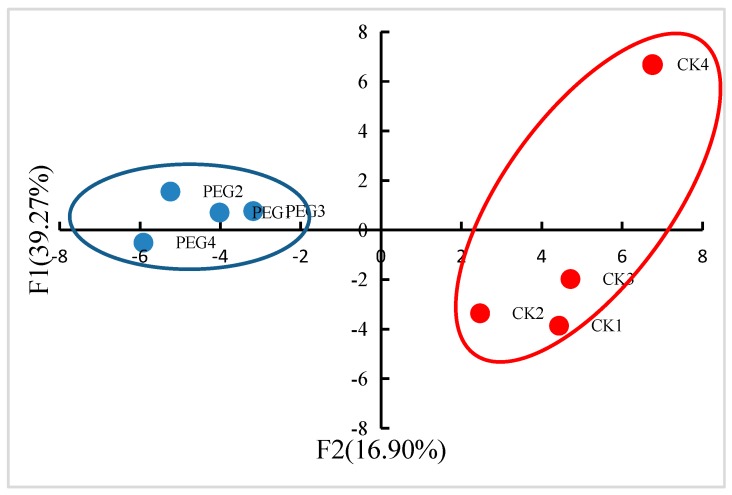
Principal component analysis (PCA) of wheat under drought for 4 days and controls.

Correlation analysis was conducted between the four parallel replicates (Figure 2). The correlation coefficient between every two replicates was more than 0.98, indicating the excellent biological repeatability of protein expression. PCA was also performed to characterize DEPs in the four replicates of the two treatments here. As Figure 3 showed, samples in two treatments were separated into two correctly, indicating that drought stress caused a significant difference in protein expression.

Comparative proteome analysis was carried out in the seedling leaves of cultivar Jinmai 47 under drought stress and well-watered conditions. 60,109 unique spectra were generated. Of them, 33,561 unique peptides can be matched to 5437 proteins. Among them, 3932 proteins were identified quantitatively (Table S1). 176 proteins presented significant differences in their accumulation levels, compared between the two water conditions, at a relative ratio of >1.2 or <0.83, *p* < 0.05 (Table S3). 65 (36.9%) proteins displayed declined accumulation, while 111 (63.1%) proteins showed increased levels under drought stress.

### 2.3. Functional Annotation, Classification and Subcellular Localization of DEPs under Drought Stress

Functional annotation of 176 DEPs was conducted using the Blast2GO program against the non-redundant (nr) NCBI database (Viridiplantae database (txid: 33090, sequence: 5104920)). After that, the proteins were mapped to the pathways in the Kyoto Encyclopedia of Genes and Genomes (KEGG) database (Table S2). Of 176 DEPs, 70 were enriched in 62 pathways, mainly involved in metabolism pathways such as biosynthesis of antibiotics (20), phenylpropanoid biosynthesis (15), starch and sucrose metabolism (10), galactose metabolism (8), glutathione metabolism (7), glycolysis/gluconeogenesis (6), cyanoamino acid metabolism (6), drug metabolism - cytochrome P450 (6), metabolism of xenobiotics by cytochrome P450 (6), and thiamine metabolism (6).

**Figure 4:**
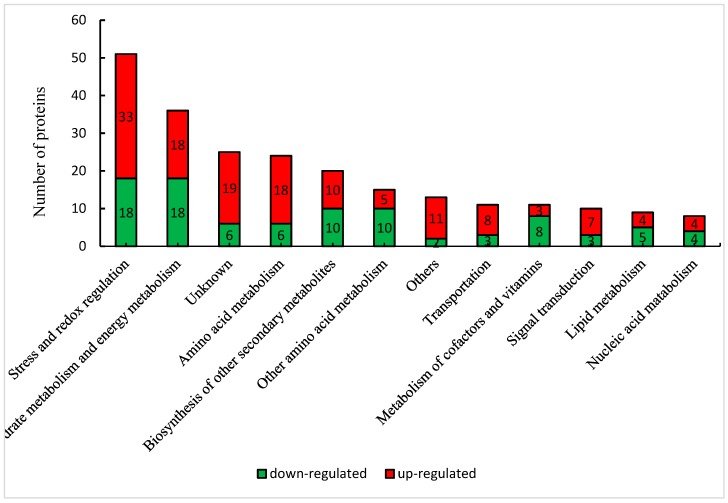
Functional classification of DEPs under drought stress.

To gain complete functional information of all 176 DEPs, we classified them on the basis of the NCBI database. All DEPs were classified into 13 categories (Figure 4). The largest category was stress and redox regulation (51), followed by carbohydrate metabolism, energy and photosynthesis metabolism (36), amino acid metabolism (24), biosynthesis of secondary metabolites (20) and unknown proteins (25).

The stress and redox regulation category also ranked first in the number of up-regulated DEPs (33). LEA protein family of this category increased most significantly, with nine family members, including 11 kDa LEA protein (A0A3B6TPI1), salt-induced YSK2 dehydrin 3 (A0A3B6QMR3), Group2 LEA protein (Q8LP43), LEA protein (A0A3B5Y269), rab protein (A0A3B6PTJ6), LEA14-A (A0A3B6GU21), LEA protein (A0A3B5Y545), dehydrin 5 (A0A3B6QLV9), LEAprotein 31 (A0A3B6TU18) displayed 1.3 to 2.53 fold of that in CK. In addition, 2 chaperone protein ClpDs (A0A3B6MVC4, A0A3B6LR88) increased by 1.82 and 1.55 fold, respectively. For antioxidant proteins, however, 4 peroxidases (PODs) (A0A3B6B862, A0A3B5YTC1, A0A3B6SFH1, A0A3B6MX35), 2 peroxiredoxins (PRXs) (A0A3B6SJF8, A0A3B6RN44), 1 probable glutathione S-transferases (GST) (A0A3B6G148), and 1 ferritin (A0A3B6LKC3) increased slightly, far less than some LEA proteins. Furthermore, catalase (CAT, A0A3B6NJS8), 2 PODs (A0A3B6MKF9, A0A3B6ET60) decreased significantly. The number of up-regulated DEPs in amino acid metabolism category ranked second. This category was characterized by a significant increase in enzymes related to proline biosynthesis. Delta-1-pyrroline-5-carboxylate synthase (P5CS, W5ACM8), P5CS2, pyrroline-5-carboxylate reductase (P5CR, A0A3B6H2J0), and ornithine aminotransferase (OAT, A0A3B6MXE9) increased from 1.21 to 1.98 fold. Carbohydrate metabolism category, including 18 up-regulated proteins, ranked the third. Proteins related to sucrose biosynthesis, sucrose synthase 4 (SUS4, A0A3B6JH89) and galactinol-sucrose galactosyltransferase (RFS, A0A3B6EFU9, A0A3B6GR62), increased 1.6 fold, 1.22 and 1.46 fold, respectively. However, those involved in sucrose degradation, cell wall invertase (INV, A0A3B6JRS1) and fructokinase-2 (A0A3B6RKI2) decreased to 0.74 fold and 0.80 fold, respectively. Pyruvate dehydrogenase E1 component subunit alpha (PDHE1α-2, A0A3B6RHJ4), citrate synthase (CS), aconitate hydratase (ACO, A0A3B6NVD9) participated in TCA cycle, and fructose and mannose metabolism were increased to 1.25–1.53 fold. Surprisingly, except for the only down-regulated rubisco activase small subunit (RACS, AOA3B6JGN7), no other enzymes related to photosynthesis showed significant changes. Other categories, including lipid metabolism, signal transduction, nucleic acid metabolism, cofactor and vitamin metabolism (thiamine metabolism and riboflavin metabolism mainly), as well as metabolisms of other amino acid (glutathione metabolism and cyanoamino acid metabolism mainly), seemed to respond relatively weakly to drought stress (Table S3).

**Figure 5:**
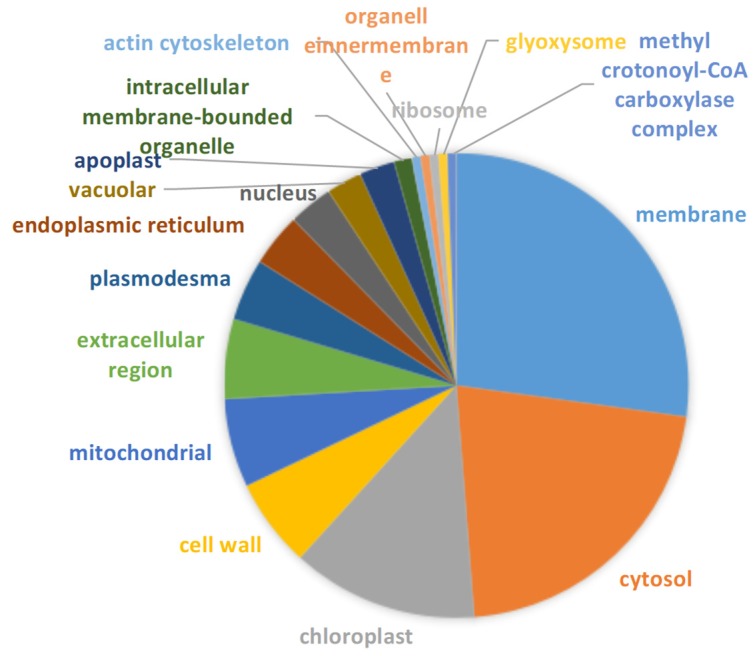
Subcellular localization of the DEPs under drought stress.

**Figure 6:**
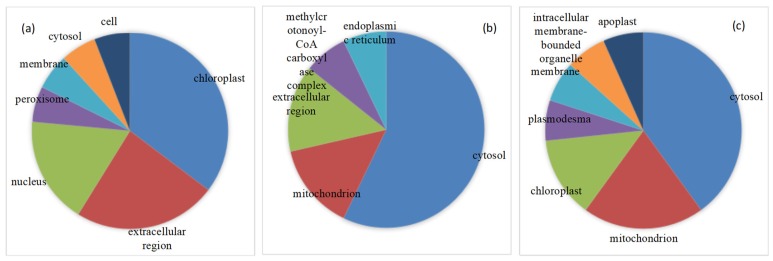
Subcellular location of up-regulated proteins involved in stress and redox regulation (**a**), amino acid metabolism (**b**) as well as carbohydrate and energy metabolism (**c**).

**Figure 7:**
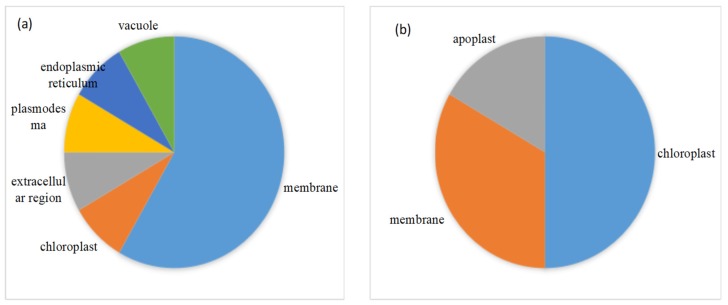
Subcellular location of function unknown up-regulated DEPs (**a**) and down-regulated DEPs (**b**).

114 of 176 DEPs were successfully annotated subcellular location. Membrane, cytosol and chloroplast were predominant regions where these DEPs were located (Figure 5). Subcellular location of proteins further analyzed aimed at the pathways in which most proteins were up-regulated. Seventeen of 33 up-regulated DEPs involved in stress and redox regulation were located in chloroplast (6), extracellular region (plasmodesma and cell wall) (4), nucleus (3) mainly (Figure 6a). Lea14-A, which functions in dehydration protection of proteins and increased by 2.53-fold under stress, was distributed in the cytosol. Fourteen of 18 up-regulated proteins involved in amino acid metabolism were located in the cytosol (8), mitochondrion (2), and extracellular region (2) predominantly (Figure 6b). P5CS2, which is involved in proline biosynthesis and increased by 1.98-fold, was located in cytosol. Thirteen of 18 up-regulated DEPs related to carbohydrate and energy metabolism were located in the cytosol (6), mitochondrion (3), and chloroplast (2) primarily (Figure 6c). The subcellular localization of 15 in 25 unknown DEPs was also analyzed. Interestingly, most increased DEPs existed in the membrane (Figure 7a), while the majority of downregulated DEPs appeared in chloroplast and membrane (Figure 7b).

### 2.4. Transcriptional Expression Analysis of Genes Encoding Several Selected DEPs

**Figure 8:**
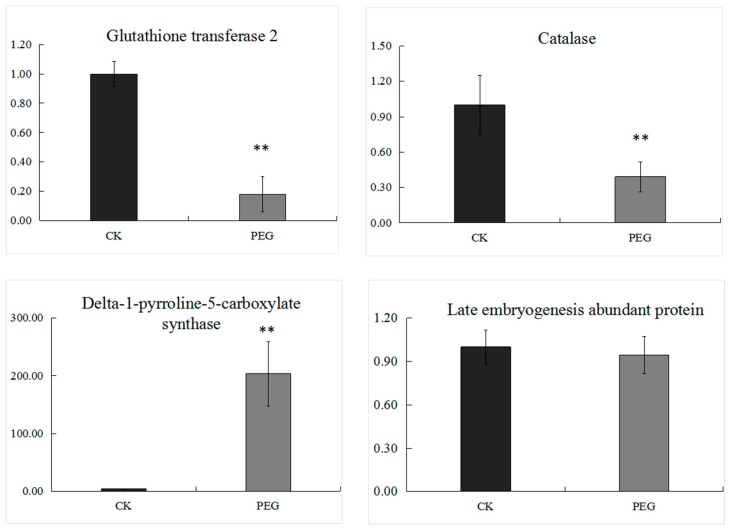
Transcript levels of selected DEPs by qRT-PCR. The data shown here are the mean ± SD of 3 biological replicates. ** represents significant difference at *p* < 0.01.

Several GSTs and a CAT, the important antioxidant enzymes, were down-regulated significantly under stress in Jinmai 47. LEA protein, a marked dehydration responsive protein; and P5CS, a crucial synthase of proline, was enhanced massively under stress. In order to reveal their expression in transcriptional levels, we measured mRNA levels of genes encoding the 4 proteins by quantitative real-time PCR (qRT-PCR) (Figure 8). GST2 and CAT were down-regulated in both transcript level and protein expression level; P5CS was up-regulated in both levels. LEA protein increased to 1.91-fold in protein level, while it showed no significant difference in mRNA level. The results showed that proline biosynthesis was enhanced, while the two antioxidant enzymes, GST and CAT decreased in response to drought. Regarding LEA protein, the poor agreement between mRNA level and protein expression level might be ascribed to the post-transcriptional regulation and/or post-translation modifications.

## 3. Discussion

The excellent wheat cultivar, which is suitable for growing in drought rain-fed areas, was subjected to a proteomics investigation after being exposed to drought stress for 4 days. The high throughput approach combined with bioinformatics analysis was hoped to comprehensively explain and deeply probe how the cultivar responds to drought stress. Here, 176 DEPs in 13 categories were obtained. 151 of the DEPs were successfully annotated function, and 114 of them were annotated subcellular localization. The highly expressed or suppressed DEPs as well as the metabolism pathways were paid more attention, in order to obtain useful information for breeding of new rain-fed cultivars.

### 3.1. Carbohydrate Metabolism and Photosynthesis

Carbon is the source of energy, and carbohydrate metabolism in organisms play an essential role in maintaining normal growth and development under stress conditions [26]. Our data showed that SUS4 in plants under drought stress increased to 1.6-fold of control under drought stress (Table S3). SUS catalyzes sucrose synthesis by transferring the glucosyl moiety of ADP glucose to the non-reducing end of an existing a-1,4-glucan chain [4]. It has been reported to be involved in the biosynthesis of sugar polymers, mainly including starch and cellulose, and generation of energy (ATP) [27,28]. Moreover, UDP-glucose-4-epimerase (A0A3B6A3S2) and RFS, which participate in the biosynthesis of raffinose and sucrose, were promoted under drought. On the other hand, INV and fructokinase-2, which associate with sucrose degradation, declined under stress. INV plays a key role in primary metabolism and plant development by hydrolyzing sucrose into glucose and fructose [29,30]. Responses of these DEPs involved in carbohydrate metabolism suggest that wheat genotype Jinmai 47 strongly enhanced sugar biosynthesis when subjected to drought stress. Elevating sucrose under stresses is a common strategy to relief damage, since sucrose can play its role as a compatible osmolyte and can have a protective effect on protein stabilization [31].

On the other hand, TCA was enhanced under drought in Jinmai 47. Three proteins involved in TCA cycle and fructose and mannose metabolism—CS, PDHE1α-2 and ACO—were up-regulated under drought stress, in agreement with previous research on wheat [4]. Enhancing TCA cycle activity may provide energy for diverse metabolisms towards tolerance of drought stress. Regarding photosynthesis, many photosynthesis-related proteins declined under stress, as reported by previous studies [4,11]. In the present study, however, only RACS was suppressed. The small subunit is key and speed-limit in photosynthesis, as proved by Andrew’s study in transgenic tobacco (*Nicotiana tabacum* W38). They found that hemizygous leaves with a single antisense gene directed against rubisco’s small subunit had 35% of rubisco content of control leaves, and the CO_2_ assimilation rate was reduced to 40% of that in controls [32]. The fact that no other proteins about photosynthesis were down-regulated might demonstrate the strong drought resistance of Jinmai 47. Another possibility is that most down-regulated members in the unknown protein category were located in the chloroplast (Figure 7b); those suppressed proteins might be photosynthesis-related enzymes. These unknown proteins remain to be further studied. In terms of carbohydrate metabolism and photosynthesis, Jinmai 47, after exposure to drought stress for 4 days, was distinctly characterized by enhanced sucrose biosynthesis and TCA cycle.

### 3.2. Amino Acid Metabolism

**Figure 9:**
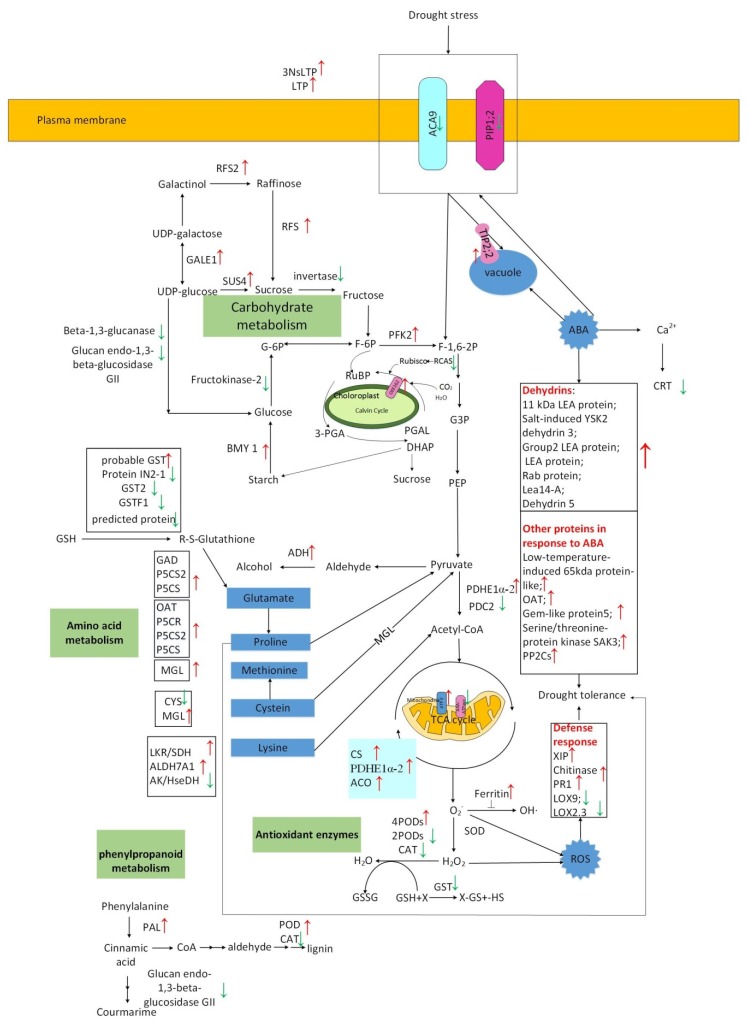
A pathway model of drought stress responses in Jinmai 47 seedlings. Abbreviations: NsLTP: non-specific lipid-transfer protein; LTP: lipid transfer protein; ACA9: probable calcium-transporting ATPase 9; GALE1: UDP-glucose 4-epimerase 1; RFS: probable galactinol-sucrose galactosyltransferase; PIP2-5: aquaporin PIP2-5; TIP2;2: probable aquaporin TIP2-2; SUS4: sucrose synthase 4; Invertase: cell wall invertase; PFK2: ATP-dependent 6-phosphofructokinase 2; PDHE1α-2: pyruvate dehydrogenase E1 component subunit alpha-2; PDC2: pyruvate decarboxylase 2; BMY1:beta-amylase1; OEE162: outer envelope pore protein 16-2; RCAS: rubisco activase small subunit; probable GST: probable glutathione S-transferase; GST2: glutathione transferase 2; GSTF1: probable glutathione S-transferase GSTF1; GAD: Glutamate decarboxylase; ADH4: alcohol dehydrogenase-like 4; P5CS2: delta-1-pyrroline-5-carboxylate synthase 2; P5CS: deltal-pyrroline-5-carboxylate synthetase; OAT: ornithine aminotransferase; P5CR: pyrroline-5-carboxylate reductase; MGL: methionine gamma-lyase; CYS: beta-cyanoalanine synthase; LKR/SDH: lysine-ketoglutarate reductase/saccharopine dehydrogenase1; ALDH7A1: aldehyde dehydrogenase family 7 member; AK/HseDH: bifunctional aspartokinase/homoserine dehydrogenase 2; PAL: phenylalanine ammonia-lyase; ATP e: ATP synthase E chain; AAA-ATPase: AAA-ATPase ASD; CS: citrate synthase; PDHE1α-2: pyruvate dehydrogenase E1 component subunit alpha-2; ACO: aconitate hydratase; PODs: Peroxidases; CAT: catalase; SOD: superoxide dismutase; XIP: xylanase inhibitor protein 1; PR1: pathogenesis-related protein 1; PP2Cs: protein phosphatase 2C; LEA protein: late embryogenesis abundant protein; LOX9: linoleate 9S-lipoxygenase; LOX2.3: lipoxygenase 2.3.

Many amino acid pathways in plants participate in regulation of tolerance and adaption to stresses. In this study, proteins involved in metabolisms of glutamate, proline (Pro), methionine (Met), cysteine (Cys), and lysine (Lys) changed significantly. P5CR, P5CS2, P5CS and OAT, involved in important pathways for Pro biosynthesis, were significantly higher under drought stress. P5CS2, P5CS and glutamate decarboxylase (GAD, D8L9S2) were related to glutamate degradation. Up-regulation of these enzymes accelerated glutamate reduction. Methioninegamma-lyase (MGL, A0A3B5YXW9) here increased by 1.58-fold under stress treatment. Most cellular Met is converted to S-adenosyl-Met, which was used in essential plant processes such as synthesis of ethylene, cell walls, chlorophyll, DNA replication as well as secondary metabolites. However, *AtMGL*, methionine homeostasis gene *Methionine gamma-lyase* in Arabidopsis, catabolizes Met in an alternative pathway, resulting in the synthesis of Isoleucine (Ile) [33]. Ile, similar to Pro, accumulate as osmoprotectants under drought [34]. The increase of MGL here suggested the decrease of Met and accumulation of Ile (Figure 9), but no direct evidence of the elevation of Ile was found. Rebeille [35] reported that the up-regulation of *AtMGL* occurred in response to concurrent biotic and abiotic stresses. The decrease of Cysteine synthase (CYS, D6QX85) and the increase of MGL, which function in biosynthesis and degradation of Cys here indicate that Cys biosynthesis might be impaired under drought stress. Moreover, lysine-ketoglutarate reductase/saccharopine dehydrogenase1 (LKR/SDH, A0A3B6NTI8) and aldehyde dehydrogenase family 7 member (ALDH7A1, A0A3B6LKM3) related to lysine degradation increased under drought, while bifunctional aspartokinase/homoserine dehydrogenase 2 (AK/HseDH, A3B6LKX6) involved in lysine biosynthesis decreased, implying that lysine accumulation was suppressed under stress. In terms of amino acid metabolism, Jinmai 47, after being subjected to drought stress for 4 days, enhanced conversion and degradation process of some amino acids, including glutamate, Met, Cys and Lys, but strongly improved synthesis process of Pro, which can also be proved by the transcriptional expression increase of gene encoding of the key proline synthesis enzyme P5CS (Figure 8). Similar to sucrose, Pro, as a compatible osmolyte, plays an important role in protecting protoplasm from dehydration [36].

### 3.3. Stress and Redox Regulation

Plants enhance antioxidant systems to protect cells against the damage caused by high levels of ROS under stress conditions. However, in Jinmai 47 seedlings under drought stress, though most antioxidant enzymes were up-regulated, such as 4 PODs (A0A3B6B862, A0A3B5YTC1, A0A3B6SFH1, A0A3B6MX35), few of them were highly expressed. Moreover, 2 PODs (A0A3B6ET60, A0A3B6MKF9) and CAT were reduced. Even the common antioxidant enzyme, superoxide dismutase (SOD), did not significantly differ from the control. Blue copper proteins (BCPs), which were found to mediate response and tolerance to Aluminum stress and oxidative stress [37,38,39], decreased to 0.5 to-0.6- fold of control. GSTs, participating in the detoxification of xenobiotics and limiting oxidative damage, were down-regulated, also declining in transcript level (Figure 7). However, a probable GST (A0A3B6G148) was 1.47-fold up-regulated. Zhu et al. reported the contrast expression of GST8 and GST6 under ABA [22]. Ferritin, serving as a regulatory role in iron storage and homeostasis and contributing to plant resistance responses [40], was 1.36-fold up-regulated. The reaction between ferrous iron and H_2_O_2_ could result in the formation of ·OH, the most dangerous ROS under osmotic stress [41]. Thus, the increased ferritin here contributes to neutralization of ROS-induced damage. To conclude, in Jinmai 47 seedlings under drought stress, the antioxidant system did not respond as strongly as expected, except that some enzymes, such as 4 PODs, a probable GST, and ferritin were increased to play protective roles against ROS.

In comparison, LEA proteins in Jinmai 47 seedlings strongly increased in response to drought stress. Nine up-regulated proteins increased by 1.3 to 2.53 fold, with Group 2 LEA protein (Q8LP43), LEA protein(A0A3B5Y269), Rab protein (A0A3B6PTJ6), and Lea14-A (A0A3B6GU21) rising by 1.88, 1.91, 2.51, and 2.53 fold of that in CK. This result is in agreement with previous researches. Several wheat sequences encoding group 2 LEA proteins have been isolated mainly from cDNA libraries’ investigative tissues of stressed seedlings. These genes were induced by cold [36,42], dehydration [43,44], salt [45], and ABA [46]. However, transcript analysis of LEA protein gene showed no significant difference in mRNA level between stress and CK, differing from the 1.91-fold increase of protein level. The poor agreement between mRNA level and protein expression level was ascribed to post-transcriptional regulation and/or post-translation modifications. Three mechanisms that help LEA proteins protect plant cells against abiotic stress have been revealed—binding of metal ions or lipid vesicles [47,48], hydration or ion sequestration [49], and, remarkably, stabilization of proteins and membranes in adaption to abiotic stress. The other function is the protection of proteins against heat-induced inactivation or aggregation under stress conditions [50,51]. Besides, Caseinolytic proteases (Clps) perform important roles in removing protein, which aggregates from cells and can otherwise prove to be highly toxic [52]. Over-expression of rice ClpD1 protein enhanced tolerance to salt and desiccation stresses in transgenic Arabidopsis plants [52]. In this study, ClpD1 increased by 1.82-fold (A0A3B6MVC4) and 1.55- fold (A0A3B6LR88), respectively, to play its protective role under drought stress.

Thus, it can be seen that under drought stress, although the wheat genotype Jinmai 47 enhanced antioxidant systems to some extent, it spent more energy on cell stabilization and protein protection.

### 3.4. Signal Transduction

Calreticulin-3 (CRT) is an important binding protein in the endoplasmic reticulum (ER). It plays its role in cellular processes, ranging from Ca^2+^ storage and release, protein synthesis, to molecular chaperone activity [51]. Our data showed that CRT protein was impaired under drought. However, in tobacco, CRT protein was overexpressed in response to drought [46]; in Hanxuan-10, a drought-tolerant cultivar, CRT protein abundance increased significantly by 6h after stress and then decreased to a great degree [46]. The 0.77-fold CRT after PEG treatment for 4 days might reach a low level after a great increase. Purple acid phosphatases (PAPs, A0A3B6KQ30, A0A3B6LHH5) also displayed significant decrease under stress. PAPs work in ROS generation and scavenge or stress-activated signal transduction. PAPs participate in thiamine and riboflavin metabolism. Oxidative and NaCl stress, but sufficient Phosphorus, promotes the expression of GmPAP3 gene in soybean. However, in alfalfa, inactive purple acid phosphatase-like protein decreased under osmotic stress [53]. Cytochrome P450 71A1 (CYP71A1, A0A3B6KH30) here were suppressed under PEG treatment. Cytochrome P450 (CYP) takes part in the synthesis of numerous secondary metabolites that play roles as growth and developmental signals or keep plants from various biotic and abiotic stresses [54]. Plant PP2Cs have been found as regulators of signal transduction pathways, such as ABA signaling [55]. Probable protein phosphatase 2C 59(A0A3B6RL15) and probable protein phosphatase 2C 70 isoform X1 (A0A3B6MX95) were higher under drought that in CK. Therefore, most proteins playing roles in signal transduction were impaired, except for probable protein phosphatase 2C 59 (A0A3B6RL15), probable protein phosphatase 2C 70 isoform X1 (A0A3B6MX95), and serine/threonine-protein kinase SAPK3 (A0A3B5Y0E9).

### 3.5. Transportation

Outer envelope pore protein 16-2 (OEP162, A0A3B6IT89) increased significantly to 1.82 fold of CK. *OEP162* expression was reported to mediate ABA signaling in seeds. The germination of *OEP162* knockout mutant seeds showed ABA hypersensitivity and amino acid metabolism imbalance [56]. OEP162 was located in chloroplast. Thus, its high expression suggests that this enzyme may modulate amino acid transport in chloroplast under stress. Plant non-specific lipid transfer proteins (LTPs) transfer phospholipids as well as galactolipids across membranes (https://www.uniprot.org/). In this study, 4 LTPs (A0A3B6GKQ2, W5D2I6, A0A341ZAA7, A0A3B6FF82) showed significant increase under drought stress, increasing by 1.34 to 1.91-fold of CK. LTPs are involved in the transfer of lipids by the extracellular matrix to form cuticular wax. Previous researches showed that LTP3 positively regulated plant response to abiotic stresses. The overexpression of *LTP3* had enhanced the freezing tolerance of Arabidopsis constitutively [57] *LTP3* knockout mutant was more sensitive to drought stress than wild type plants [57]. In tobacco, a 6-fold increase of *LTP* transcripts was observed after three drying events [58]. Thus, under serious drought stress, Jinmai 47 might begin to improve cuticular wax formation by enhancing LTPs in order to prevent water loss as well as high-temperature damage to leaves accompanying transpiration reduction.

### 3.6. Secondary Metabolites

20 DEPs are participating in the biosynthesis of secondary metabolites, 15 of which are involved in phenylpropanoid biosynthesis. The phenylpropanoid pathway leads to the biosynthesis of lignin, flavonoids, condensed tannins and so on, all of which possess antioxidant properties in protecting plants against stresses [59]. Phenylalanine ammonia-lyase (PAL) catalyzes the first reaction in the biosynthesis from l-phenylalanine to trans-cinnamate, a precursor of various metabolites produced in response to stresses [40], and also catalyzes the reaction from tyrosine to p-coumaric acid. Here, pal accumulation was enhanced to 1.31-fold under stress. Beta-1,3-glucanase (GLB3, Q9XEN5), glucan endo-1,3-beta-glucosidase gii (D8L9Q2), beta-glucosidase 5-like (A0A3B6ERB4), and 2 predicted proteins (A0A3B6H5C6, A0A3B6H5R7) belonging to gentiobiase, which also serve as the precursor of coumarine, were decreased. Suppression of these gentiobiases under drought might lead to coumarine biosynthesis reduction. PODs and CAT here play their roles as lactoperoxidase, which catalyze the reaction from p-Coumaryl alcohol to various lignins, such as p-Hydroxy-phenyl lignin and guaiacyl lignin. Most PODs here were promoted, while CAT was suppressed. The changes in phenylpropanoid biosynthesis metabolism mainly affected accumulation of cinnamic trans-cinnamate, coumarine and lignin.

### 3.7. Other Metabolism Categories

The majority of DEPs involved in signal transduction were impaired, except probable protein phosphatase 2C 59, probable protein phosphatase 2C 70 isoform X1, and serine/threonine-protein kinase SAPK3. Signaling system might be strongly initiated at an early stage of the stress response. Here, after 4 days of severe drought stress, membrane ion leakage began to increase. The plants are supposed to be concentrating on resistance, instead of signaling to evoke a response. In addition to the signaling system, lipid metabolism, nucleic acid metabolism, cofactor and vitamin metabolism, as well as metabolisms of other amino acid seemed to show a relatively weak response to drought stress.

## 4. Materials and Methods

### 4.1. Materials and Growth Conditions

Wheat seeds were surface-sterilized by 10% sodium hypochlorite for 10 min, rinsed with distilled water for several times, and soaked in distilled water for 8 h. After soaking, they were placed on water-wetted filter papers in culture dishes to germinate. The experiment was conducted in a growth chamber with 16 h/8 h photoperiod at day/night temperature of 23 °C/20 °C. Seedlings were grown in distilled water at the same temperature cycle. After 8 days, the seedlings were transplanted in rectangular pots with 1/2 Hoagland nutrient solution for 7 days in the same environment. Similar seedlings were selected and divided into two groups. A group was cultivated in 1/2 Hoagland nutrient solution continuously (CK); the other one was cultivated in 1/2 Hoagland nutrient solution with 20% Polyethylene glycol-6000 (PEG-6000).

### 4.2. Membrane Permeability Measurements

The method of measurement of membrane permeability referred to [60]. Some minor alterations were made here. The second fully expanded leaves were detached from plants under stress or well-watered conditions and washed briefly with deionized water. The leaves for each treatment were cut into 1 cm fragments and immersed in 10 mL deionized water. They were exposed to air by pump for 30 min, followed by agitating for 3 h. Afterwards, the first conductivity was measured and recorded. The leaf fragments in bathing solution were boiled for 15 min to obtain the second conductivity (total conductivity). Three replicates were measured per treatment.

(1) $$\mathrm{Relative}\;\mathrm{Conductivity}=\frac{1\mathrm{st}\;\mathrm{Conductivity}}{2\mathrm{nd}\;\mathrm{Conductivity}}\times 100\%$$

### 4.3. Protein Extraction

Leaves of wheat treated by PEG for 4 days were harvested and frozen in liquid nitrogen and stored at −80°C. Each treatment contained 4 biological replicates. Acetone extraction method was adopted here to extract total protein from the leaves. A lysis buffer containing 7 M urea, 2 M thiourea, 0.1% CHAPS and 1% protease inhibitor cocktail was conducted in extracting proteins. After centrifugation (13000 g) for 15 min at 4 °C, centrifuge tubes with four volumes of precipitation solution (trichloroacetic acid:acetone, 1:9) were used to transfer the supernatants into, which were stored at −80 °C for 24 h. Bradford assay was used to perform quantification of proteins.

### 4.4. Protein Digestion and iTRAQ Labeling of Samples

For each sample, 100 μg protein was mixed with lysis buffer containing 25 mM DTT and 50 mM iodoacetamide. The filters were then washed three times using 300 μL dissolution buffer (20 mM triethylammonium bicarbonte), and then being centrifuged at 10000× *g* for 10 min. Subsequently, the proteins were digested at 37 °C overnight with 2 μg trypsin at a ratio of 1:50. The resulting peptides were labeled with 8-plex iTRAQ tags (ABsciex, Framingham, MA, USA), as shown as Table 1. Four replicates in 2 treatments were processed. The labeled mixture was incubated at room temperature for 2.5 h and vacuum-dried.

### 4.5. High pH Reversed-Phase (HpRP) Chromatography

The samples were combined into one tube and dried in vacuo after the labeling. Dried peptides were resuspended in 100 μL of mobile phase A. Afterwards, peptides in phase A were centrifuged at 14,000 g for 20 min. The supernatants were loaded on the XBridge^®^ peptide BEH C18 column (130 Å, 3.5 μm, 4.6 mm × 150 mm) (Waters, Milford, MA, USA. ) and eluted stepwise by applicating mobile B in the RIGOL L-3000 system (RIGOL, Beijing, China). Mobile phase A was composed of 2% (*v*/*v)* acetonitrile, 98% (*v*/*v*) ddH_2_O and pH 10, and Phase B consisted of 98% (*v*/*v*) acetonitrile, 2% (*v*/*v*) ddH_2_O and pH 10. Peptides were eluted by 5% mobile B for 10 min, 5–20.5% mobile B for 21.7 min, followed by 20.5–95% mobile B for 15.3 min, and returned to 5% mobile B for 4 min finally at a 1 mL/min flow rate. The fractions were eluted at 1-min intervals and collected using step gradients of mobile B. 40 fractions were collected and pooled into 10 final samples, which were dried using a vacuum centrifuge.

### 4.6. Mass Spectrometer Analysis

LC-MS/MS analysis was performed on an Orbitrap Q-Exactive-plus mass spectrometer (Thermo Fisher Scientific, Waltham, MA, USA) combined with an EASY-nLC 1000 nanosystem (Thermo Fisher Scientific, Waltham, MA, USA). The dried iTRAQ-labelled peptides fractions were dissolved in 20 μL of liquid containing 0.1% formic acid. After centrifugation at 12,000 g for 10 min, the supernatant was collected. The peptide mixtures were then separated using a binary solvent system with 99.9% H_2_O, 0.1% formic acid (phase A) and 80% acetonitrile, 0.1% formic acid (phase B). Linear gradients of 8–18% B for 20 min., 18–32% B for 85 min, 32–50% B for 28 min, 50–95% B for 17 min, with a flow rate of 600 nL/min, were employed. The eluent was input into a QE plus mass spectrometer through an EASY-Spray ion source. The mass spectrometer was adjusted automatically between MS and MS/MS mode. The full scan MS mode was operated with the following parameters: automatic gain control (AGC) target, 3e6; resolution, 70,000 FWHM; and scan range, 350–1800 m/z. The MS/MS mode was set as follows: activation type, HCD; collision energy, 32%; resolution, 17,500 FWHM; scan range, 300–1800 m/z AGC target, 1e5.

### 4.7. Database Searching and Protein Identification

All MS and MS/MS spectrum data were analyzed using ProteoWizard (version 3.0.8789). The MS/MS spectra were searched using the Mascot search engine against the UniProt-wheat_UP000019116 (130,673 entries, 8 January 2019). 2 missed cleavages, ion mass tolerance of 0.05 Da, and parent ion tolerance of 10 ppm for a peptide fragment were permitted. Carbamidomethylation at cysteine was set as a fixed modification, and oxidation at methionine was defined as a variable modification. The proteomic software Scaffold Q+ (version 4.6.2) was used for protein quality controlling and quantitation. The quantitative analysis was carried out on the log2-values of the measured intensities, and the data were normalized based on the median using Perseus software (version 1.5.5). DEPs were analyzed for significant down-regulation or up-regulation. Ratio of the abundance of the proteins identified in plants under PEG treatment to that of CK was used to assess their fold changes. Moreover, a two-sample t-test was used to identify significant (*p* < 0.05) differences in means between the two treatments. DEPs were defined on the basis of thresholds of >1.2- or <0.83-fold change ratios and *p* value < 0.05 in plants under PEG treatment compared to those of CK. The false discovery rate (FDR) of peptides was less than 1.0%. At least two specific peptides should be identified in each protein, and normalized by median data.

### 4.8. Functional Annotation and Analysis of DEPs

Functional annotation of proteins was conducted using the Blast2GO software against non-redundant (nr) NCBI databases (Viridiplantae database (txid: 33090, sequence: 5104920)). Kyoto Encyclopedia of Genes and Genomes (KEGG) pathway analyses were performed on DEPs.

### 4.9. qRT-PCR Analysis

Total RNA was extracted from leaves of wheat with RNeasy Plant Mini Kit (Qiagen, Inc., Hilden, Germany ) under 20% PEG for four days and that of control plants. DNA contamination was removed with an on-column DNase (Qiagen, Inc.) treatment. IScript cDNA synthesis kit (BioRad Laboratories, Hercules, CA, USA) was used here for 1 mg of total RNA to reverse transcribe into first strand cDNA in a 20 μL reaction. cDNA was then diluted into 10 ng/μL, 2 ng/μL, 0.4 ng/μL and 0.08 ng/μL concentration series. An ABI 7500 Sequence Detection System from Applied Biosystems (Applied Biosystems, Singapore) was adopted here for three replicates of real-time PCR experiments for each concentration. GAPDH served as the reference gene here. The primers for target genes were designed by Primer Express software (Applied Biosystems) and the sequences are shown in Table 2. No primer dimmers or false amplicons interfered with the result since primer titration and dissociation experiments were performed. Ct number was extracted for both reference gene and target gene with auto baseline and manual threshold after the real-time PCR experiment.

### 4.10. Statistical Analysis

The data were subjected to T-test with SAS software. We use * and ** to present there are significant differences between plants under stress or well-watered at the levels *p* < 0.05 and *p* < 0.01, respectively. Microsoft Excel was used to plot the results.

## 5. Conclusions

The proteomics analysis of wheat genotype Jinmai 47 revealed some of its drought response characteristics. The antioxidant system was not highly expressed as expected. Expression of photosynthesis-related proteins was not affected significantly by drought treatment, except rubisco activase small subunit. TCA cycle related proteins were up-regulated, which may enhance energy supply for multiple biological processes to resist stress. The wheat genotype Jinmai 47, when subjected to severe drought stress, seems to be characterized by substantial enhancement in plasma and protein protection mechanism. Improving sucrose and Pro biosynthesis, the important compatible osmolytes, contributes to stabilizing cells by protecting plasma from dehydration under drought stress. Pro biosynthesis was proven in mRNA levels as well. The high expressions of LEA proteins and chaperone proteins play essential roles in safeguarding proteins when dehydrated. However, qRT-PCR analysis showed that the mRNA level of LEA protein had a poor agreement with its protein level, which might be due to post-transcriptional regulation or post-translational modification. An excellent rani-fed cultivar, which can stably gain higher yield in rain-fed areas, must be capable of tolerating more severe and longer drought, and promptly recovering growth after release from stress, or even keeping photosynthesis to some extent under stress conditions. The robust protective mechanism might not only improve drought resistance, but also ensure strong and prompt recovery. Based on this study, SUS4 (1.60), P5CSs (1.34, 1.98), OAT (1.22), Rab protein (2.51), and Lea14-A (2.53) were considered to be important candidate proteins, which deserve to be further investigated.

## Figures and Tables

**Table 1:** Four replicates in 2 treatments were labelled by different iTRAQ regeants.

NO	113	114	115	116	117	118	119	121
1	CK1	CK2	PEG1	PEG2				POOL1
2			CK3		CK4	PEG3	PEG4	POOL2

**Table 2:** The primer sequences used in qRT-PCR.

Accession Number of Protein	Name of Protein	Accession Number of Related Gene	Primer Sequences (5′-3′)	Product Length (bp)
I7FHT3	Glutathione transferase 2	TraesCS1D02G190000	F: GCCCGTGCTCATCCACAA	220
			R: CAGCCCCTCCGCCTTCT	
A0A3B6NJS8	Catalase	TraesCS6A02G041700	F: CCCAAACTACCTGATGCTCC	203
			R: TGATCCTCGTCTTCTCCCTTC	
A0A077RXE4	Delta-1-pyrroline-5-carboxylate synthase	TraesCS3B02G395900	F: ACCCTGAAGGCTGGAAAGATA	176
			R: GCATCAGGACGAGACTCAAAA	
A0A3B5Y545	late embryogenesis abundant protein	TraesCS1A02G364000	F: GGACCAGACCGCCAGCAC	261
			R: CCCATGCCCAGCGTGTT	
W5GYX5	GAPDH	TraesCS6D02G196300	F: GTTTGGCATTGTTGAGGGTT	268
			R: ATCATAGGTTGCTGGCTTCG	

## Supplementary Materials

Supplementary materials can be found at https://www.mdpi.com/1422-0067/20/11/2621/s1.

## Abbreviations

DEP	Differentially expressed protein
ROS	Reactive oxygen species
PEG	polyethylene glycol-6000
iTRAQ	Isobaric tags for relative and absolute quantitation
PCA	Principal component analysis
KEGG	KyotoEncyclopedia of Genes and Genomes
qRT-PCR	Quantitative real-time PCR
